# A longitudinal multi-site evaluation of community-based partnerships: implications for researchers, funders, and communities

**DOI:** 10.1186/s12961-023-01045-y

**Published:** 2023-10-03

**Authors:** Virginia J. Lewis, Catherine M. Scott, Kate Silburn, William L. Miller

**Affiliations:** 1 Australian Institute for Primary Care and Ageing, La Trobe University, Bundoora, VIC 3086 Australia; 2 Department of Community Health Sciences, University of Calgary, Calgary, AB Canada; 3 K2A Consulting, Calgary, Canada; 4 Department of Family Medicine, Lehigh Valley Health Network, Allentown, PA USA; 5 University of South Florida Morsani College of Medicine, Tampa, USA

**Keywords:** Community academic partnerships, Community-based participatory research, Multi-sectorial research partnerships, Longitudinal qualitative evaluation, Implementing innovative interventions

## Abstract

**Background:**

Innovative Models Promoting Access to Care Transformation (IMPACT) was a five-year (2013–2018), Canadian-Australian research program that aimed to use a community-based partnership approach to transform primary health care (PHC) organizational structures to improve access to appropriate care for vulnerable populations. Local Innovation Partnerships (LIPs) were developed to support the IMPACT research program, and to be ongoing structures that would continue to drive local improvements to PHC.

**Methods:**

A longitudinal development-focused evaluation explored the overall approach to governance, relationships and processes of the LIPs in the IMPACT program. Semi-structured interviews were conducted with purposively selected participants including researchers with implementation roles and non-researchers who were members of LIPs at four time points: early in the development of the LIPs in 2014; during intervention development in 2015/2016; at the intervention implementation phase in 2017; and nearing completion of the research program in 2018.  A hybrid deductive-inductive thematic analysis approach was used. A Guide developed to support the program was used as the framework for designing questions and analysing data using a qualitative descriptive method initially. A visual representation was developed and refined after each round of data collection to illustrate emerging themes around governance, processes and relationship building that were demonstrated by IMPACT LIPs. After all rounds of data collection, an overarching cross-case analysis of narrative summaries of each site was conducted.

**Results:**

Common components of the LIPs identified across all rounds of data collection related to governance structures, stakeholder relationships, collaborative processes, and contextual barriers.  LIPs were seen primarily as a structure to support implementation of a research project rather than an ongoing multisectoral community-based partnership.  LIPs had relationships with many and varied stakeholders although not necessarily in ways that reflected the intended purpose. Collaboration was valued, but multiple barriers impeded the ability of LIPs to enact real collaboration in daily operations over time. We learned that experience, history, and time matter, especially with respect to community-oriented collaborative skills, structures, and relationships.

**Conclusions:**

This longitudinal multiple case study offers lessons and implications for researchers, funders, and potential stakeholders in community-based participatory research.

## Overview of the study

### Background

Current implementation research places a strong emphasis on working collaboratively across multiple sectors to address complex health and social questions (e.g., [7, 9]). Literature argues for the value of stakeholders working together in partnership to design and implement a wide range of interventions (e.g., [2, 4]), but there are few studies that evaluate the processes of developing and sustaining multisectoral community-based partnerships in detail over time, and those that do tend to rely on retrospective recall of participants and focus on single projects and partnerships (e.g. [1]).

*Innovative Models Promoting Access to Care Transformation* (IMPACT) was a 5-year (2013–2018), Canadian–Australian research program that aimed to use a community-based partnership approach to transform primary health care (PHC) organizational structures to improve access to appropriate care for vulnerable populations. From the outset, the researchers recognized that these organizational level interventions would require “meaningful partnerships between researchers, decision-makers, care providers and community representatives” (proposal p 4) and plans were made to operationalize this multisectoral collaborative approach through Local Innovation Partnerships (LIPs). The LIPs were seen as mechanisms for sustaining successful interventions after the IMPACT grant finished, and enduring local structures that could continue to develop and implement innovative ways to improve access to PHC.

A developmental evaluation approach was considered particularly appropriate for supporting adaptive learning and continuous improvement throughout implementation of the IMPACT innovations [3, 6]. While not part of the initial impact proposal, this approach to evaluation of the LIPs was planned to document progress and challenges and to offer real-time feedback to facilitate the operationalization and collaborative processes of the LIPs. It was necessary to adapt the approach to reflect some of the realities of this multi-site research program. For example, the Principal Investigators (PIs) at each site were the primary contact people for members of their LIP. It was not feasible for the evaluators (VL, CS) to collaborate directly with local LIPs to use the results to conceptualize and redesign local innovation. While the evaluators shared the results with PIs during each data collection and analysis cycle, they were not actively involved in supporting use of the results to inform decision-making at the local level. The exception to this was author CS who also led a local LIP. Throughout the evaluation, the evaluators maintained a commitment to generating and sharing results to support learning and adaptation of the interventions. Using a longitudinal, multiple cross-case evaluation design, we explored the differences, similarities, and evolution of LIPs across three Canadian and two Australian IMPACT teams working to design, implement, and evaluate local, organizational interventions to improve access to primary health care for vulnerable populations.

In this paper we:

- Describe what was intended as a community-based partnership approach to service design, delivery and evaluation of the IMPACT sites.
- Describe how Local Innovation Partnerships (LIPs) unfolded across sites in terms of governance structures, stakeholder relationships and collaborative processes.
- Explore the implications of findings for funders, researchers, clinical service providers, and community members who want to implement interventions through community-based partnership.

## IMPACT research program plan

A full description of the IMPACT research program is published elsewhere [8]. The four objectives of the research program were to:

1. To develop a network of partnerships (LIPs) between decision makers, researchers and community members to support the improvement of access to PHC for vulnerable populations.
2. To identify organisational, system-level community-based primary health care innovations designed to improve access to appropriate care for vulnerable populations and establish the effectiveness and scalability of the most promising innovations.
3. To support the selection, adaptation and implementation of innovations that align with regional partners’ local populations’ needs and priorities.
4. To evaluate the effectiveness, efficiency and further scalability of these innovations.

**Fig. 1:**
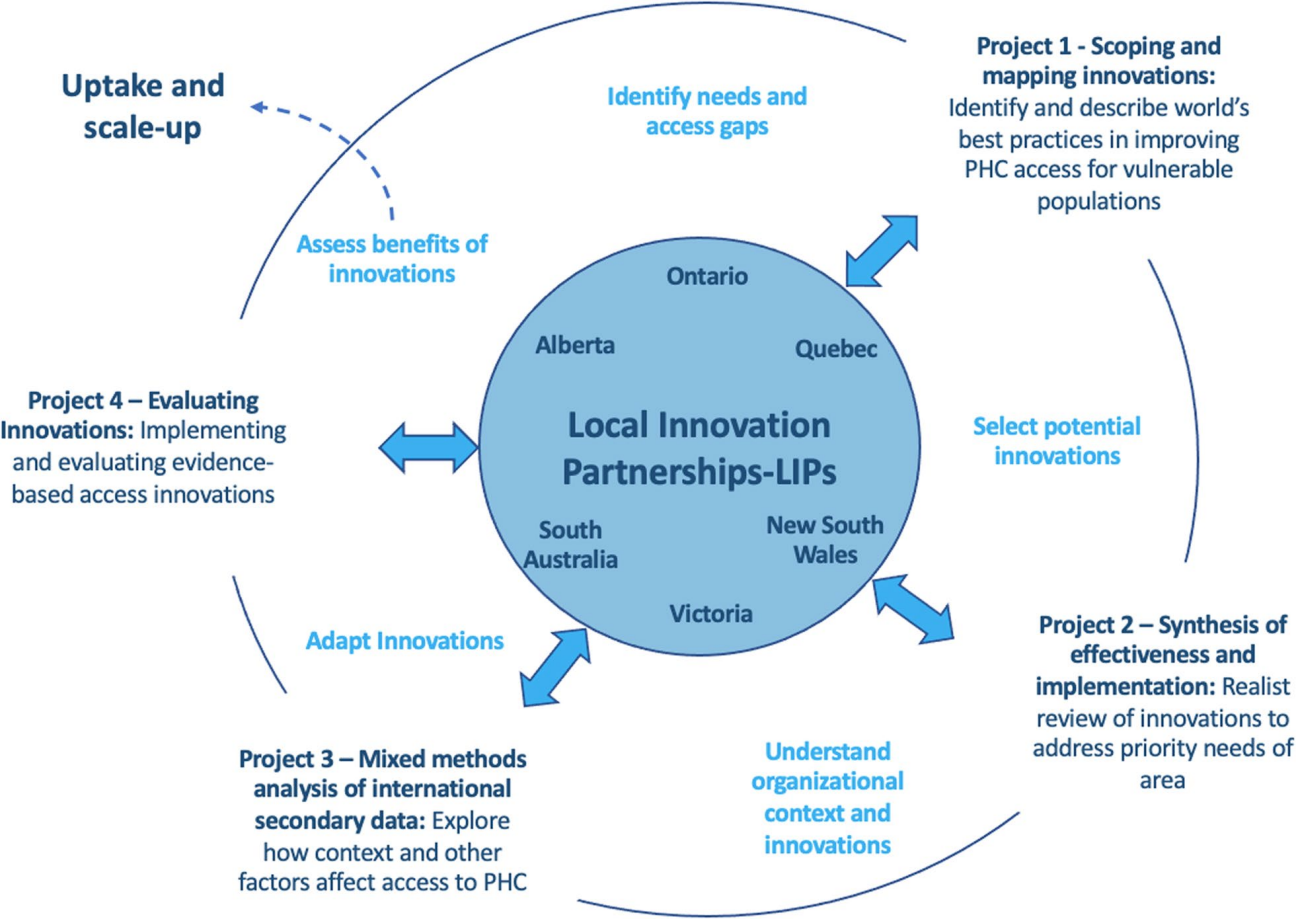
The overall design of the IMPACT program. Adapted from Russell et al. [8]

The IMPACT LIPs were established in communities with complex challenges to the delivery of community-based primary health care (PHC). There were six original LIP sites in IMPACT. One of the original six sites (South Australia) was not included in the evaluation presented here as it ceased to operate a LIP following a system-level restructure after the first year. Figure 1 illustrates the centrality of the LIPs in the overall design of the IMPACT program of research. LIPs were defined as “a community of stakeholders who share a common concern around vulnerable populations that are at increased risk due to limited access-to-care.” The IMPACT program aimed to “generate sustainable, national and international communities of practice able to generate innovative solutions to hitherto intractable access barriers to appropriate PHC for vulnerable populations”.

LIP responsibilities were to:

- characterise the local health system context
- identify the accessibility needs of the local population related to PHC and the most vulnerable subgroups
- undertake a collaborative process of decision making through a series of deliberative processes including forums
- combine local knowledge with the evidence emerging from the program of empirical research to design innovative models of care
- implement and adapt an organisational intervention
- collect data using common tools and procedures wherever possible
- support evaluation of access to PHC for the selected vulnerable populations.

Early discussions highlighted the different levels of experience of working in community-based partnerships among the IMPACT researchers, including Principal Investigators (PIs) and research staff at each site. Therefore, a resource was prepared in 2014 to support the development of LIPs and enable meaningful engagement, knowledge exchange and collaboration among stakeholders as they carried out research program activities. The Principles and Procedures for the Local Innovation Partnerships Implementation Guide (hereafter, the Guide) [11] provided evidence-informed advice about how LIPs could be developed and run. While the Guide was endorsed by the research program governance group (IMPACT international executive committee, composed of the principal investigators and international project manager) to support a common approach, it was not a set of mandatory instructions as it was expected that there would be variation across settings.

The LIP was conceived as “the foundation for a set of collaborative activities” (p. 6). As such, the Guide provided guidelines for development of LIPs. In addition to the LIP responsibilities listed above, it described the role of the LIP in “anchoring the research program in the local community through deliberative processes for public consultation and collective decision-making” (p. 8) The Guide included “how-to” advice on governance, engagement, and evaluation of collaboration.

In terms of governance, the Guide included the advice that LIPs would likely need multiple distinct structures including a small group with five-eight people including researchers and non-researchers to oversee local implementation details, a broad LIP network of stakeholders to provide strategic advice and feedback, and an optional layer between these two groups to support resource mobilization for implementation. It was recommended that researchers, local decision-makers, clinicians, and community members be included in all structures. Key responsibilities were outlined for the role of “LIP coordinators” who were part of the research team at each site. Developing a Charter and Memorandum of Understanding (MoU) to support clear delineation of roles and responsibilities in the partners was recommended and templates were provided. The Guide also outlined steps for clarifying context, setting engagement objectives, identifying key stakeholders, considering appropriate approaches to engagement, developing a communication plan to support transparency within the partnership, and sustaining stakeholder involvement over time (pages 12 and 13). A high-level timeline of activities for LIPs was also provided.

## Methods for a developmental approach to evaluation

A longitudinal development-focused evaluation was designed to explore how the overall approach to governance, relationships and processes in the IMPACT program supported each LIP to design, develop, implement, and sustain interventions. This developmental evaluation focused on the role of the LIPs and was separate to the process and impact evaluation of the interventions. The LIP evaluation was intended to provide information to inform ongoing implementation of the LIPs throughout the research program. Findings were presented to the international executive committee at annual meetings and to the individual PIs for each LIP site. We used a longitudinal, multiple cross-case design with qualitative data collected from stakeholders at multiple time points using semi-structured interview schedules.

**Fig. 2:**
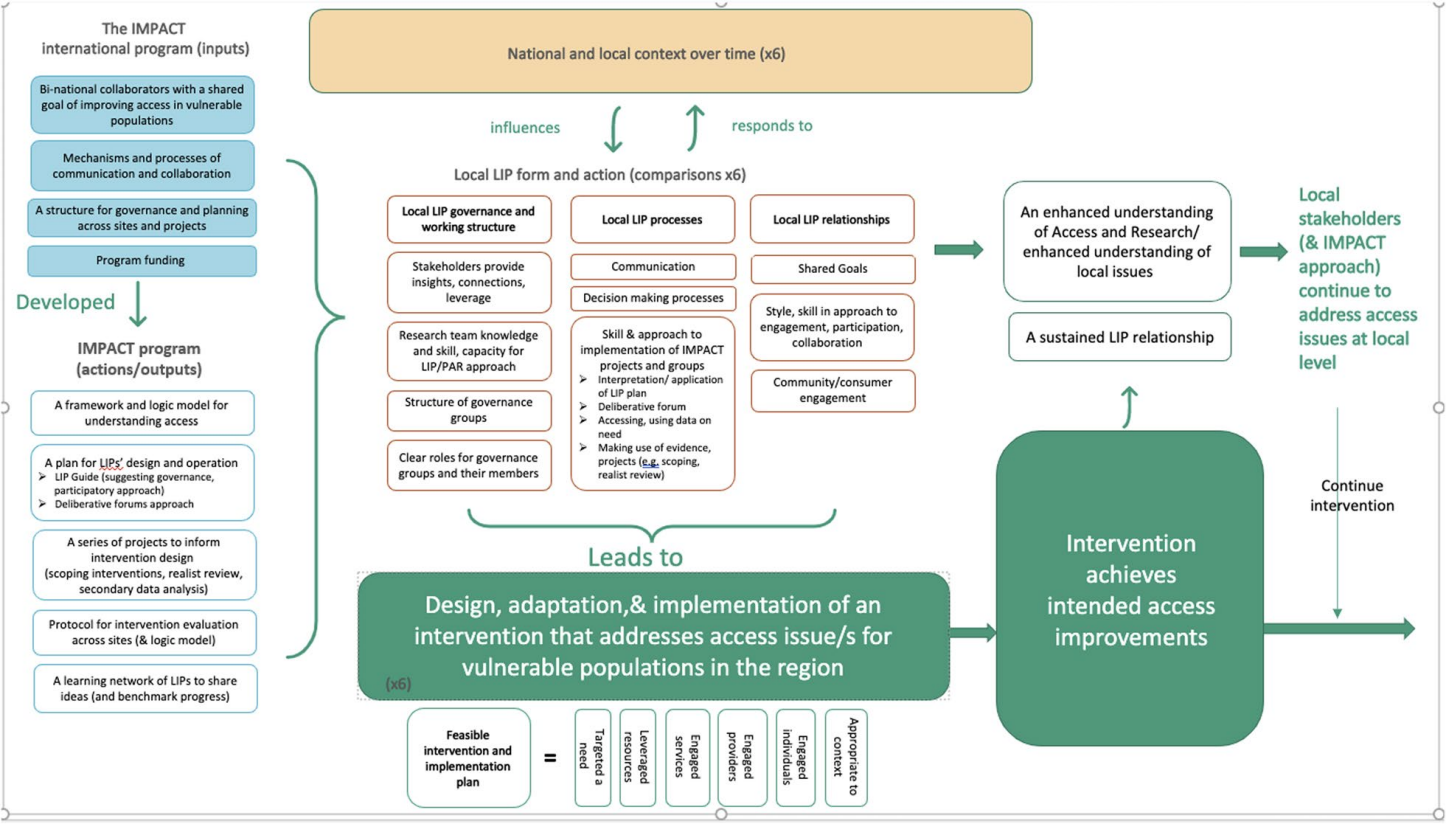
Representation demonstrating the relationship between research projects, LIPs and interventions including LIP form and action domains

Interviews were conducted at four time points, early in the development of the LIPs (2014); during intervention development (2015/2016); at the intervention implementation phase (2017); and nearing completion of the program of research (2018). Interview guides were developed based on the Guide and an evolving visual representation that placed the LIPs in the context of the overall research program and focussed on capturing the development of their form and function (see Fig. 2). Interview guides were updated by the evaluation leads (CS, VL) prior to each evaluation phase in response to emerging findings and feedback from previous rounds of data collection and analysis.

Individual participants were purposively selected to provide insight into each of the LIPs based on their roles and experience at the time of the interviews. At each round, participants included the national project managers, LIP PIs (also called LIP Leads), LIP coordinators, and additional researcher LIP members at each site. In addition, in 2017, two or more non-researcher members of the LIPs were interviewed for four LIPs. These individuals were nominated by the researcher team members. Individual interviews were conducted by telephone and using online meeting platforms. In total, 18 participants were interviewed in 2014, 14 in 2015/2016, 34 in 2017 (of which 15 were non-researcher LIP members), and 30 in 2018 (including 12 non-researcher members).

We used a hybrid deductive-inductive thematic analysis approach. The deductive aspect of the analysis approach came from using the initial LIP Guide as the framework for designing questions and analysing responses. The inductive aspect came from using the results of each round of data collection over the five years to continue to develop and describe the IMPACT approach. A visual representation was developed and refined after each round of data collection to illustrate emerging themes around governance, processes and relationship building that were demonstrated by IMPACT LIPs (Fig. 2). Interviews were recorded and analysed after each round of data collection by the evaluation team members. This included immersion in the data by reading and re-reading notes and listening to transcripts repeatedly to summarise the way interviewees described the LIP using a qualitative descriptive method [5, 10] aligned to the interview questions.

**Fig. 3:**
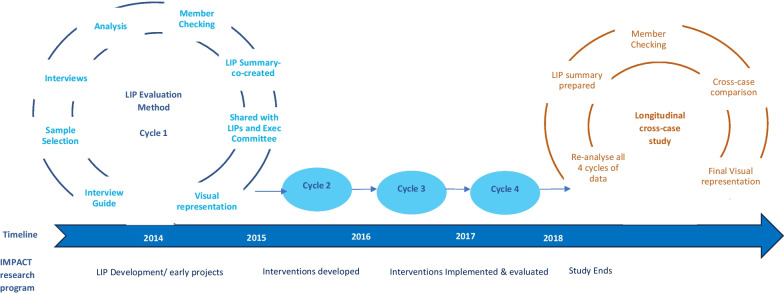
Visual representation of data collection and analysis phases

After completion of all rounds of data collection, all available texts and transcripts were re-analysed to develop narratives at the level of each LIP. A structured coding technique was used initially, based on a-priori concepts identified from the interview and research questions, and emergent concepts identified during the analytic review process. This coding approach was informed by the evaluation protocol, the Guide, and the evolving visual representation of IMPACT (Fig. 2). Two researchers independently coded a sample of interviews to confirm inter-coder reliability. Similarly coded segments were then aggregated for more detailed coding and inductive interpretation and the analysis documented. The validity of this analysis was confirmed by a senior investigator external to the program (WLM), who reviewed a sample of coded data and the documented analysis. Narrative summaries covering the whole period of the project (2013–2018) were prepared for each LIP and were sent to researchers at each LIP for member checking. Following finalisation, the external senior investigator analysed the narrative summaries to identify over-arching, cross-case codes and themes which form the basis of the interpretations in the next section. Figure 3 visualizes the methods described above. In referring to examples that demonstrate findings, the five LIPs included in the data analysis are labelled A1 to A2 for two Australian sites, and C1 to C3 for three Canadian sites in some places, and by the researcher/non-researcher label in others. A date is provided where relevant.

## Analysis and interpretation of developmental evaluation data

The common components of the LIPs identified across all rounds of data collection and analysis (reflected in Fig. 2) related to governance structures, stakeholder relationships, collaborative processes, and contextual barriers.

### Governance structures

Through cross-case comparison, it is evident that there was variation in approaches to governance across LIPs and a lack of clarity about governance structures and the roles of stakeholders in most of the LIPs. Furthermore, interviewees from the same LIP (researchers and non-researchers) often provided different descriptions of structures and processes compared to other members of the same LIP. From the outset, governance and working structures of each LIP varied and most continued making further adaptations during the 5 years. Although each site could describe a group that brought together researchers and non-researchers, one common variation from the initial proposed structure was that all the LIPs described a “Research Team” as a key group in their governance structures. This group generally included the investigators and staff employed by the grant and, in most cases, was distinguished from the Core Team by an absence of non-researchers. Most interviewees made a distinction between operational day-to-day tasks and decision-making, and generally saw the Research Team doing the former and the Core Team the latter; however, feedback from interviews suggested the roles were not this clear-cut and non-researchers were excluded from key decision-making in most LIPs (see “processes” below).

Use of formal agreements to articulate roles and responsibilities varied across sites. Two of the five sites did not establish formal MoUs to support the governance structures but developed Terms of Reference (ToR) for groups involving non-researchers. One of these also had a Charter that was reviewed regularly, including updating roles and responsibilities. Two others had agreements in place with pre-existing groups that involved community-based organisations. One site described no form of MoU or ToR being established. Where there was an MoU, it did not necessarily serve the function of delineating roles and responsibilities. As one researcher commented: ‘the MoU does not really define the responsibilities of the Core Group, just that we are going to work together…’ [Researcher, A1, 2014].

Most Core Team groups met monthly to bi-monthly in the early years. Over time, the groups with non-researchers met less frequently and became less connected to the work in most sites. This contributed to further lack of role clarity. For example, in 2015 one team reported that the Core Team was taking a less active role than before, described as hearing reports about the project and making occasional suggestions to the realist review (A1, 2015). By 2017 interviewees in this LIP described the Core Team as having an oversight and approval role, but the IMPACT research team had not engaged with or reported to them for 12 months (A1, 2017). Another team described their Core Team as having an important hands-on perspective, but they did not meet for 18 months between 2016 and 2017 (C2, 2017). Non-researchers on this team reported feeling disconnected from the project, not sure about whether their involvement was required, and unclear of their roles in the Core Team. There were contrasting views about the extent to which the researchers were reporting to the Core Team versus asking for guidance. One researcher noted that “we report regularly to [the Core Team] but in reality, they don’t have much oversight” (A2, 2014). One non-researcher team member said: ‘I don’t think my responsibility [in the Core Team] was ever articulated’ (A1, 2018).

Despite the differences in approach to use of formal agreements and frequency of formal meetings with the Core Teams, roles and responsibilities remained unclear for most interviewees throughout the rounds of data collection. Overall, the cross-case comparison suggested the LIPs were seen primarily as a structure to support implementation of a research project rather than an ongoing multisectoral community-based partnership intended to drive improved accessibility of primary health care for vulnerable populations in their local communities over the longer term. Within this limited scope, the feedback from non-researchers was generally positive, with frequent references to good will as the motivation to maintain their involvement despite the lack of clarity about roles and responsibilities.

### Stakeholder relationships

It was acknowledged in the Guide that there were different kinds of stakeholders relevant to the successful operation of the LIP, and LIPs would have changing relationships with people during the implementation of the research program. Interviewees from one site noted that they had good representation of local organisations and involved new people as required, as new ongoing members or on an ad-hoc basis: “rather than try to have all of the perspectives on the core team [we] would have ad-hoc members who have particular interests and expertise [and who could be invited] when there was content relevant to them” (C1, 2015). The international nature of the IMPACT program was a strong positive factor in engaging non-researchers from policy and health service delivery settings. For example, Australian non-researcher stakeholders reported that their interest in participating was linked to their interest in hearing about experiences related to access to PHC from Canada and vice versa.

There were varying perspectives on how to involve health service users or people who represented the populations being targeted by interventions in the form and function of the LIPs. There was confusion about the definition of ‘consumers’ in relation to representation on the LIPs’ governance structures. Discussion occurred at different times about whether service users should be engaged or whether the focus of collaboration should be on service providers as “consumers” of the interventions, given IMPACT was intended to be implementing organizational level interventions. One non-researcher noted that neither community members nor individual general/family practitioners (who were expected to implement the intervention) had been actively engaged (A2, 2018).

Several Canadian LIPs engaged people who might access the interventions in governance and reported that it was a positive experience and useful to the intervention design. Others were hesitant to do so for a variety of reasons. One team noted that engaging service users in governance before the focus of the intervention was determined might raise expectations that could not be met, so they focused on community consultation in the early phase of the program and looked to patient groups to be engaged in governance later (C2, 2014). Another Australian LIP thought that engaging vulnerable groups required significant time and resources and shouldn’t be done as a superficial thing (A1, 2015). Most teams recognized the importance of capturing the perspective of service users and targeted groups at some level, and although they did not involve them in governance through LIP structures, they found other ways to incorporate their perspectives. For example, some engaged community-based organisations that worked directly with vulnerable populations, rather than the people who would access services themselves (C1, A2); others used brief interactions/consultations such as focus groups (C2), and others worked with existing patient groups (C2, C3).

Engagement of a broader set of local stakeholders in a third layer of governance was described in the Guide. The focus was on involving these stakeholders in initial deliberative processes and then keeping them informed to support potential for sustainability and spread. The types of stakeholders in this group included organisations and individuals with an interest and/or role in supporting access to comprehensive primary health care and population health in the local area. While there was good engagement in the initial deliberative processes in most LIPs, most of these stakeholders were not directly involved in the interventions selected by each LIP. Despite recognition in the project proposal that it was important to keep these stakeholders informed, and some rhetoric about offering webinars and regular updates to broader networks, none of the narrative summaries described a “communication plan” for this engagement. Where some activities were described, it was not clear that the audience for them went beyond the immediate core group team members and a non-researcher noted there had been little engagement of broader stakeholders after sending an update 18 months previously (A2, 2018).

Overall, the LIPs had relationships with many and varied stakeholders although not necessarily in ways that reflected the intended purpose and outcomes described in the proposal and the Guide.

### Collaborative processes

The focus of each LIP was to design, adapt and implement an intervention to access issues for vulnerable populations in their region using information arising from the discrete projects that were part of IMPACT. The partnership evaluation focused on the collaborative processes around LIP functioning. Nonetheless, interviewees often noted that processes for collaborating around common IMPACT projects tended to be more clearly articulated than those around LIP functioning, although there was still some variability in how things were done.

Each site was able to perform the activities associated with the common projects including identifying the needs of vulnerable populations related to PHC access through deliberative processes; designing an access-focused intervention informed by evidence; and supporting implementation and evaluation of the intervention. However, the extent to which each site was able to anchor the research projects in the local community through collective decision-making by LIPs as intended varied. For example, some LIP interviewees saw the Deliberative Forums as a task to be checked off and others described them as an important collaborative process, but without a clear idea of how to use the information. Some researcher and non-researcher stakeholders described the Forums as generating a lot of ideas about access issues and ideas for addressing them but causing frustration by having no avenue to pursue them beyond the single research project focused on by the IMPACT team. This was seen to cause some non-researcher stakeholders to disengage. Similarly, realist reviews were conducted to guide design of the intervention at each site but there was little consideration of processes that would support interactions between the realist review team and the LIP Core Team so that emerging findings could be used in the short-term to help shape the intervention.

In terms of collaborative processes adopted by LIPs, one team, as an exception, reported a positive collaborative environment within the LIP Core Team from the outset, with clear communication and strong participation in discussions and decisions: “Very effective – we have bashed around ideas with each other, are free to disagree with each other, are productive, get stuff done, have a lot of laughs; all very committed to the work.” (C1, 2014)**.**

Interviewees frequently articulated the value of collaborating with non-researchers in the Core Team. Core teams involving researcher and non-researcher members were described as helpful for engaging organisations and gaining resources and facilitating stakeholder involvement in the early stages of the program. Researchers described the Core Teams as valuable in helping to design interventions that were more feasible and/or more appropriate; several commented that the Core Team had answers and ideas that the research team would not have come up with alone. Actual engagement of the multisectoral Core Teams was weaker than their articulated value. In some LIPs, collaborative processes were not used to actively involve non-researcher stakeholders in decision-making processes, with the Core Teams acting more as advisory groups than co-developers and owners of the intervention. Core Teams that did stay active during the intervention implementation and evaluation phases tended to remain involved in problem solving for day-to-day operations and ‘finding solutions’ to challenges with their interventions. The way the LIPs were described by researcher and non-researcher interviewees reflected the dominance of IMPACT as a “research project”, rather than being about trying to integrate service provider operational norms or create new innovative and sustainable structures to address PHC access issues. While each could value the other’s perspectives, there was frustration in the level of collaboration achieved expressed by interviewees in the case summaries.

Overall, collaboration was valued, but multiple barriers impeded the ability of LIPs to make the collaborative real in daily operations over time. One LIP had some success.

### Contextual barriers

Despite the intent to implement a community-based approach, IMPACT international and national governance groups and coordinating staff in each country, provision of the Guide and associated training, and the development of a visual depiction of the emerging approach each year, the evaluation revealed an overall lack of common understanding, commitment, and skills to do the multisectoral partnership work in line with the bold goals. The dominance of a traditional research mentality prevailed. There were several contributing factors to this.

#### Researcher attitudes and skills

The cross-case comparison suggested that the motivations, attitudes, and skills of the researchers for working in partnerships had an influence on the approach to organising and running LIPs. There was variable experience among researchers for working through multi-sectoral community-based partnerships, and some reluctance to develop or adapt existing structures and established approaches to managing research projects in line with the recommended IMPACT approach. For example, some researchers felt that the Core Team had taken the intervention in a different direction to what they had expected, and they felt unable to say no. One research interviewee indicated that while they appreciated the opportunity to give ‘a voice to the people to decide what they want’ they were concerned about not being able to influence decisions based on their knowledge of research evidence (C3, 2014). These comments suggest that some researchers found it difficult to share power and agency with other stakeholders and lacked strategies and processes to create a balanced approach that recognised different perspectives.

These challenges were amplified by changing research staff (particularly LIP Coordinators) and Core Team members over time, and were also dependent on how Research Teams were constructed and what types of people were engaged to advance the work. On some teams, knowledge and skills related to governance, engagement and collaborative processes were missing. Some teams were able to fill these gaps by using a consulting model, bringing in specialists as required, but others struggled to find someone with the broad range of skills or experience required for community engagement.

#### Non-researcher attitudes and skills

Non-researcher partners also described barriers to participating in a collaborative research project. Although interviewees described some positives of being associated with the international IMPACT project, such as added credibility for their interest in intervening to improve access to PHC, the goal of a “LIP Learning Network”, as described in the protocol [8], was not achieved. Researcher and non-researcher participants noted that timing of some of the required steps was not in line with the realities of conducting developmental work on the ground, and some of the information (e.g., the realist review) was generated too late or too early to be useful. Many interviewees felt that the data collection requirements that came with being an international program and a cross-site study were onerous and/or not relevant to their LIP, and it was difficult for some research teams to perceive or explain the potential value of having common data across diverse interventions. Some non-researchers also described the process of developing and implementing the intervention as slower and more resource-intensive than would be the case if it were not research.

#### Pre-existing relationships and histories

Most of the sites used pre-existing relationships to some extent to set up the LIPs which was seen as aligning the new project with initial conditions and context. Only one of the sites decided actively against using existing partnership groups and sought to build new ones based on identifying organisations and individuals relevant to the access issue.

There were some positive impacts of building on existing relationships in terms of starting from a base of trust and good will. One researcher reported:

A lot of people involved at the start of the LIP had existing relationships with [the lead researcher] which gave them a store of goodwill to build on – ‘I’ll give you the benefit of the doubt, because I’ve worked with you before and I know you are good for it’. If we had been starting fresh with relationships … we would have had a much harder time” (C1, 2015).

However, in some LIPs, the ongoing lack of clarity about the structure of the LIPs and roles for non-researchers resulted in people falling back on relationships of convenience and past histories. In some cases, these previous relationships created constraints around the scope or direction of work and limited how new members could be engaged, in comparison with the potential benefits of forming a new group and setting new expectations and implementing new ways of working.

#### Competing expectations

The stakeholder descriptions of IMPACT as a research project supported by LIPs may have undermined the likelihood that LIPs would be sustained after the project finished. Practical incompatibilities surfaced between a community-based partnership approach to undertaking research versus a grant-funded researcher-led approach.

The influence of research timelines and the amount of time required for each component of the approach were common issues described by interviewees. For example, a researcher interviewee commented that the Research Team’s commitment to valuing everyone’s opinion meant that timelines could be difficult to meet and there could be some frustration with this although it resulted in the ‘final product [being] way better than if just one person took it on’ (C1, 2015.) Many teams described feeling a sense of urgency associated with time-limited grant funding that resulted in LIPs being established before there was agreement about structural purpose and basic criteria. This resulted in rushed or skipped foundational work on partnership norms, roles, expectations, purpose, etc. and was evidenced in the difficulties LIP members had in describing their governance structures in all years of the evaluation. Perceived time pressures were also associated with a lack of willingness to revisit the way the LIPs were organised, despite the funding being for 5 years. On the other hand, one site that set norms and expectations about the purpose and functioning of the LIP demonstrated comfort with being participatory and responsive from the outset. The lead researcher in this LIP had years of experience working in a community-based collaborative way based on a strong knowledge of theory and practice-based evidence.

Overall, we learned that experience, history, and time matter; especially with respect to community-oriented collaborative skills, structures, and relationships, and the ability to manage the complexities of participatory community work.

## Implications

Through the IMPACT grant, the teams sought to implement interventions to improve PHC access for vulnerable populations while, simultaneously, trying to build local multi-sectoral community-based partnerships in five different contexts. The partnerships were intended to actively identify, implement and evaluate those interventions, and persist after the grant to further improve access by sustaining, spreading and scaling-up successful interventions. Doing research and building sustainable community-based partnerships at the same time was bold and ambitious; the experience was exciting, but also quite sobering. Our longitudinal multiple case study, which was specifically intended only to evaluate the processes of LIP development and maintenance, offers lessons and implications for researchers, funders, and potential stakeholders in community-based partnerships.

### Implications for researchers

Our experience highlights the complexity of implementing a project using a community-based partnership approach that builds new organizational structures while also developing and implementing interventions. IMPACT was fundamentally a research grant with the LIPs intended to focus on developing an appropriate and innovative intervention to improve access to PHC. The potential for the LIPs to be sustainable beyond the IMPACT research grant period was limited by the funding being held by the researchers. This contributed to the LIPs being seen as research partnerships rather than sustainable community entities.

Many skills are needed to advance a community-based partnership approach to research, including partnership development, governance, participatory decision-making, facilitation, change management, and evaluation. Each of these requires a strong understanding of concepts and extensive experience to practice well. Availability of these kinds of skills has implications for how a project is designed, managed, staffed and funded. Many research teams would not have the skills required to navigate these competency areas. Additional support will often be required and may need to come from private consultants or individuals with relevant experience who work for partner organizations or in other research areas. Contracting and managing additional resources and personnel external to the research setting is a further challenge.

Researchers and those encouraging community-based partnership approaches to research need to acknowledge time as a challenge. Given that most researchers and community partners do not have experience with these approaches, significant time must be invested at the beginning of projects to get everyone on the same page about terminology, expectations, success criteria, governance, participatory processes and more. As illustrated by the IMPACT research program, the importance of this foundational work cannot be overstated. Without intentional work to set and commit to these standards and establish a common operating framework, aspirations to take a community-based partnership approach can quickly fade leading to reversion to traditional research approaches that are more in line with simple consulting and advisory approaches. The need for the Guide became apparent early in the establishment of the research program; but it was too late to influence the LIPs as fully as it might have and was not used optimally across all sites. Recognizing this, the authors of this paper obtained a Scale and Spread grant from the Canadian Institutes of Health Research to develop a comprehensive Handbook which builds on our experiences and will serve as a guide for others who are committed to engaging with community to generate contextually-relevant primary health care improvement [12].

Even with intentional timely up-front work, regular reinforcement, monitoring and ongoing capacity building is required to ensure adherence to the approach. The many partner check-ins required to stay on course can feel awkward and time-consuming to those unfamiliar with working in this way, and sometimes even to those who *are* familiar with working in this way. It takes great diligence to operate in a participatory way over an extended period and to ensure that all stakeholders feel valued and have true co-ownership over the project.

One of the biggest factors influencing how the community-based partnership approach was implemented in the IMPACT project was the attitude and skills required to be responsive to community needs and ideas in a way that recognizes the potential value of different experiences and kinds of knowledge. Researchers cannot put aside their commitment to using evidence and rigorous methodological design; however, clear and frequent discussions about the scope of influence for all partners are needed. For IMPACT, the scope for the roles of researcher and non-researcher partnerships to consider issues of access to PHC was different to the scope for influencing how the research was designed and implemented. In a researcher-driven partnership approach, it takes time to process input fully and generate new creative solutions that are then communicated in a way that provides a clear rationale demonstrating how partners’ views were considered and decisions made. Stronger, and early, articulation of goals, roles, and responsibilities across the IMPACT team and within each LIP may have helped. Some of the questions researchers should consider (and described in more detail in the Collaboration for Spread Handbook) include:

- What is the purpose of engaging with non-researchers? Is it to support a research project through providing advice or is it more than that (e.g., making budgetary decisions)?
- Where are the skills to support effective partnering/collaboration located? Do people with specialist skills and experience need to be brought in to support the partnership?
- Do we have time to work in partnership or are the timelines and demands of the research project (and funder) likely to undermine true collaboration?

### Implications for funders

Most funding agencies now request partnerships and/or community-based participation as a condition for grants. On the other hand, researchers have to deal with the possibility that the work they put into establishing partnerships prior to securing funding may not be followed through if the funding is not granted. As illustrated by the IMPACT experience, requiring a list of partners or in-kind contributions at the point of application will not guarantee a robust collaborative approach will be used in implementing the project. Funders rely on research teams to implement these approaches, sometimes without understanding what is really required: how long it takes, what resources are required, what success criteria look like, and how to monitor or hold teams accountable for the approach. We recommend that funders carefully consider the justification for requiring partnerships and collaborative approaches, including definitions, criteria, expectations, funding, time, and target outcomes for these approaches. Without explicit justification, support and accountability structures, is it reasonable or ethical to require community-based partnership approaches?

### Implications for service providers and community members

Active participation of people who will be affected by implementation of innovations, and who are working to address similar issues to those the partnership is focused on is a requirement for effective community-based research partnerships. The experience of IMPACT highlighted the importance of being clear about roles and responsibilities of all partners. Service providers and community members who are approached to participate in design, implementation and evaluation of interventions are encouraged to carefully consider the implications of their participation. This includes assessing their potential roles in different phases, particularly where the partnership’s ultimate goal is to undertake research. Some of the questions for consideration include:

- Is this project aligned with my/my organization’s/my community’s goals?
- What are the implications of the potential outcomes of the project for me/my organization/my community?
- What level of participation is required?
- Is it realistic to participate at the required level?

To respond to such questions, service providers and community members must receive sufficient information from the research team and have the preliminary conversations to ensure that there is common understanding regarding the level of participation required over time.

We recommend that service providers and community members gather the necessary information to familiarize themselves with the project and only accept involvement if they feel that there is genuine benefit to them, their organizations/community and to the people to whom they provide services. Based on the IMPACT experience we encourage non-research partners to advocate for monitoring and evaluation of the partnership so that it is more likely to be responsive to their needs and worth the investment of their time and energy.

## Next steps

When community-based partnerships are desired and/or required for research grants, we strongly recommend that funding includes commitment to longitudinal monitoring and evaluation of the functioning of the partnership using a developmental approach to support the structures, relationships and processes to be responsiveness to context throughout the program of research.

In addition to support for monitoring and evaluation, our longitudinal evaluation suggests that investment in building capacity for collaborative ways of working (i.e., in partnership) is essential to ensure that researchers have the competencies and capability to meaningfully engage in research with communities.

Although the IMPACT experience revealed challenges, we often witnessed moments of creativity and potential breakthrough in the way researchers understood and approached partnership. When researchers, funders, clinical service providers, and community members pay heed to the questions we raised, we argue that community-based partnership research has the potential to contribute to improvements in access to primary health care.

## Data Availability

The data that support this study cannot be publicly shared due to privacy reasons; interviewees are potentially identifiable. If necessary, selected de-identified data could be made available under strict confidentiality conditions upon reasonable request to the corresponding author.

## Abbreviations

IMPACT: Innovative Models Promoting Access to Care Transformation
LIPs: Local Innovation Partnerships
PHC: Primary health care
MoU: Memorandum of Understanding
ToR: Terms of Reference
